# Supplementary material for: Reconstructing faces from fMRI patterns using deep generative neural networks
Source: Commun Biol. 2019 May 21;2:193. doi: 10.1038/s42003-019-0438-y (PMC6529435; doi:10.1038/s42003-019-0438-y)
Supplement: Supplementary file 1 — Supplementary Materials [file 42003_2019_438_MOESM1_ESM.pdf]

Supplementary Materials

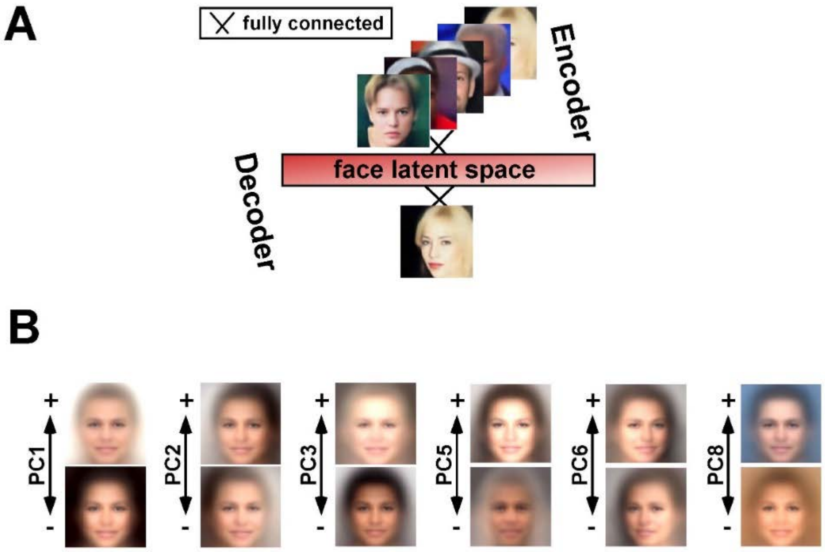

Supplementary Figure 1. PCA face decomposition model. A. Just like in our (VAE-GAN) neural network model, faces are encoded into a latent space of principal components (in red; here, for consistency with the VAE-GAN latent space dimensions, we retained only the first 1024 principal components), and can also be decoded or reconstructed from these components. The main difference, however, is that in PCA the encoding process is a simple linear combination of pixel values. **B.** Some of the first principal components reflect easily interpretable latent dimensions such as face orientation (PC2, PC6), gender (PC5, PC8), skin color (PC3) or background color (PC1, PC8).

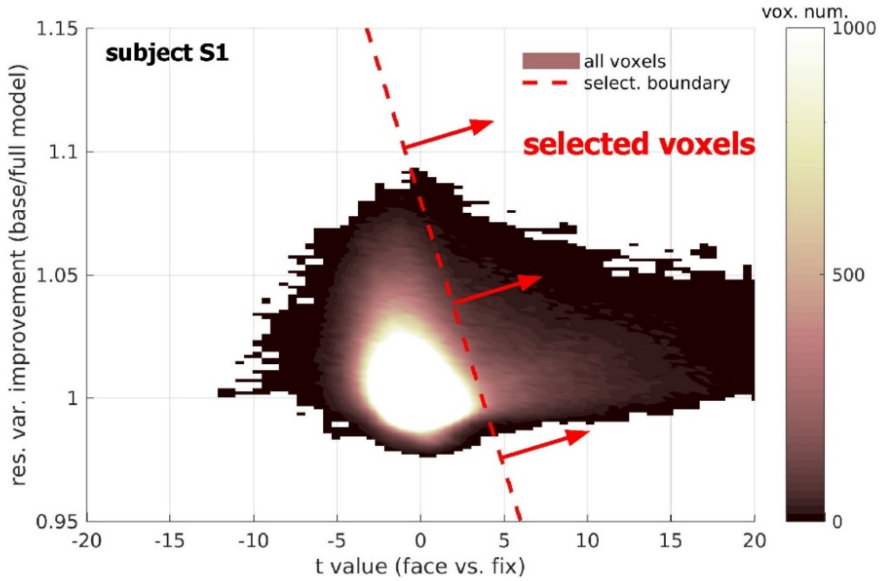

Supplementary Figure 2. Voxel selection procedure. Although fMRI-based face reconstruction was already accurate when using all of the (gray matter) voxels in the brain (about 1.3 million voxels, data not shown here), we found that performance was optimal when selecting a subset of these voxels, combining: (i) a strong visual response to the face stimuli (as measured by a t-test between the "face" condition and the "fixation conditions"; x axis); (ii) an improvement of (adjusted) residual variance when the 1024 latent face dimensions were added as parametric regressors to the baseline GLM (i.e., a GLM with a single binary regressor for face present/absent; y axis). The final selection criterion was a linear combination of these two measures (dashed red boundary). The slope of the boundary was selected, based on the voxel bivariate distribution, so that all voxels with t-values 4 and above (strongly responsive to faces) would be included regardless of their residual variance criterion; and similarly, all voxels with more than 8% adjusted residual variance improvement (strongly sensitive to face latent parameters) would be included, regardless of the t-value. This boundary was chosen based on the fMRI training data of subject S1 (illustrated here), and consequently applied to all other subjects S2-S4, thus limiting the possibility of a spuriously optimal solution. Further, to prevent "double-dipping", the voxel selection was made independently for each subject and each face encoding model (PCA or VAE-GAN), based solely on the BOLD responses collected for training, but not test images.

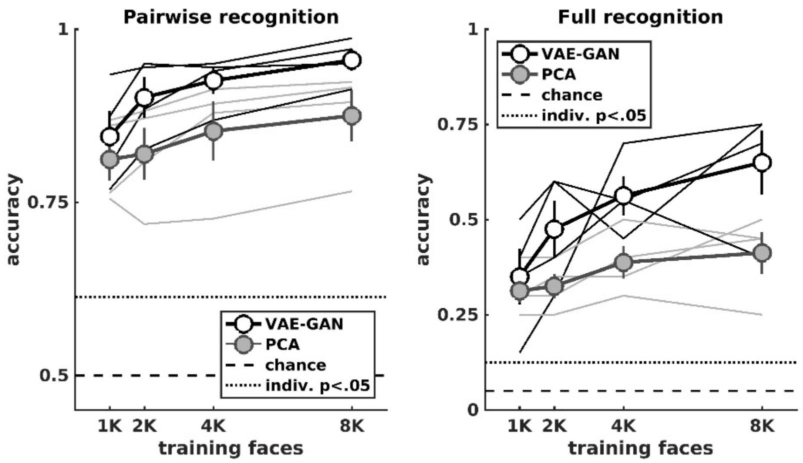

Supplementary Figure 3. Face decoding accuracy (left, pairwise recognition; right, full recognition) as a function of training dataset size. The "full dataset" performance (8 sessions per subject, amounting to ~8K faces) corresponds to the data in Figure 4B-C of the main manuscript. The brain decoding models were also trained with ~1K faces per subject (only the first session), ~2K faces (only the first two sessions) and ~4K faces (only the first four sessions). To facilitate comparison, the same voxel selection (derived using the full dataset) was applied to all training subsets. Circle symbols and thick lines represent mean (±sem) across subjects, thin lines depict individual subject data.

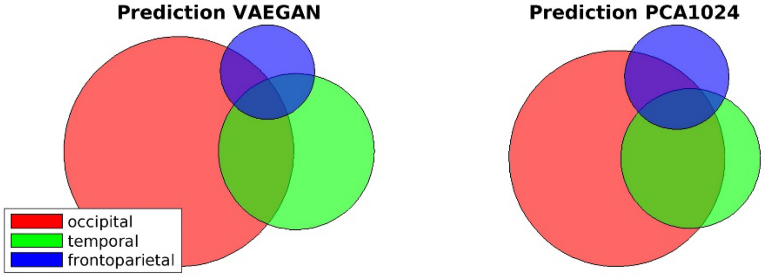

Supplementary Figure 4. Venn diagrams, averaged over subjects, of the proportion of "ground-truth" latent variable variance (for the 20 test images), uniquely or jointly explained by each of the 3 anatomical ROIs (occipital in red, temporal in green, frontoparietal in blue).

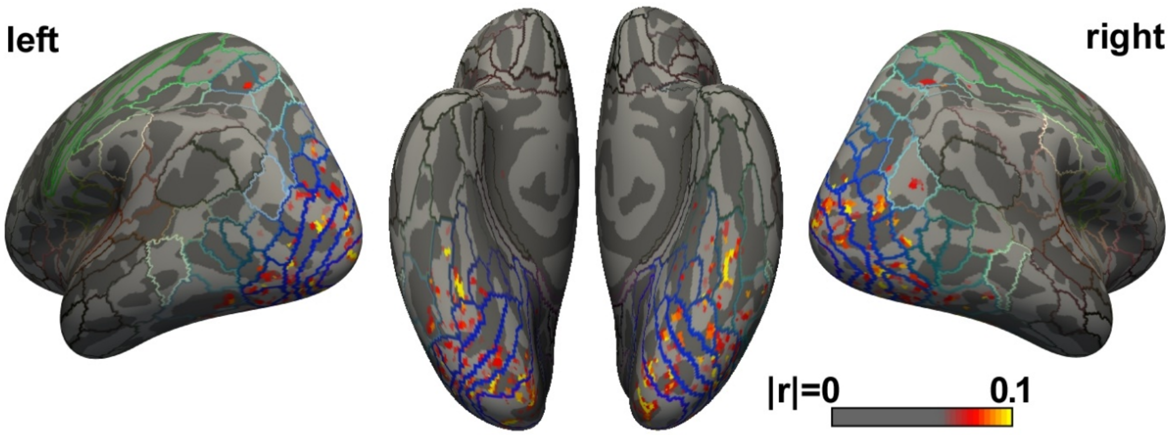

Supplementary Figure 5. Mapping of face gender-selective voxels. A "gender" latent vector was derived by subtracting the average latent description of 10,000 female faces from the average latent description of 10,000 male faces. This vector was then correlated with every column of the "brain decoding" matrix W (see Figure 2): a voxel sensitive to the "gender" property of face images should result in a strongly positive or strongly negative correlation. The subject-averaged absolute value of the correlation r is plotted here on the FreeSurfer average brain. The colored lines indicate the boundaries of standard cortical regions. Gender-selective voxels are found in early visual areas, as well as in regions of the fusiform gyrus.

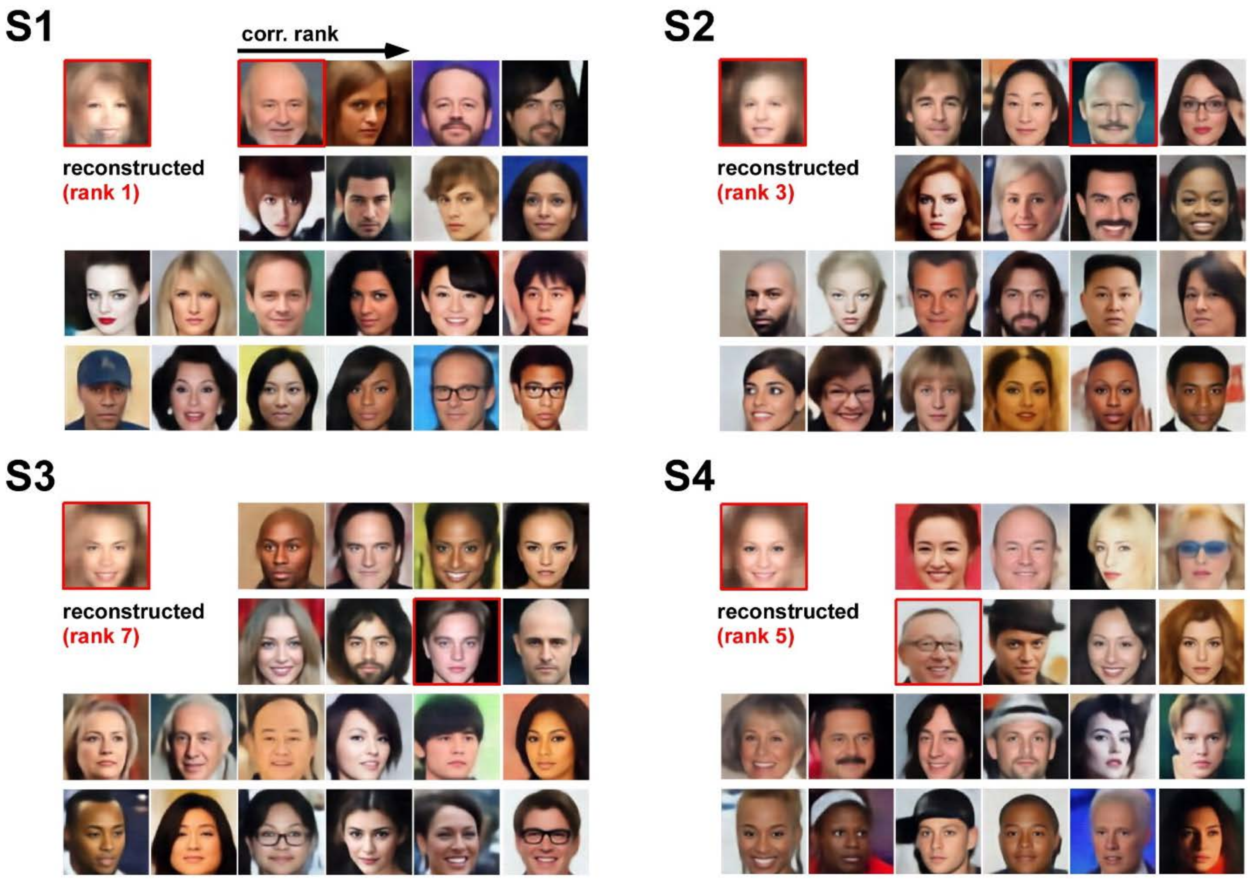

Supplementary Figure 6. Faces reconstructed from mental imagery, based on temporal voxels. For each subject, the reconstruction is shown on the top-left, followed by the 20 candidate faces, ranked by decreasing similarity with the decoded face (correlation of the latent vectors). The image chosen by the subject (i.e., the one that they imagined during the imagery trials) is highlighted in red, and its rank is also reported under the face reconstruction—the lower the rank, the higher the imagery decoding accuracy. Because the brain decoding model was trained during perception (not imagery) conditions, the faces reconstructed from imagery are much less compelling than the ones reconstructed from perception. Nonetheless, one can note interesting details in some reconstructions, such as the “mutton chops” beard for subject S1, or the shape of the eyes in subject S3.

Name	Height	Width	Channels	Kernel	Activation
Input	*(128*	*128*	*3)*		
Encoder	64	64	192	3x3	elu
	32	32	256	3x3	elu
	16	16	384	3x3	elu
	8	8	512	3x3	elu
	4	4	768	3x3	elu
	1	1	1024	fc	linear
Variational Bayes	1	1	1024	fc	-
Generator	4	4	1024	fc	elu
	8	8	512	3x3	elu
	16	16	384	3x3	elu
	32	32	256	3x3	elu
	64	64	192	3x3	elu
	128	128	3	3x3	elu
Discriminator	64	64	64	4x4	elu
	32	32	64	4x4	elu
	16	16	64	4x4	elu
	8	8	64	4x4	elu
	4	4	64	4x4	elu
	1	1	1	fc	sigmoid
Batch size	64				
Loss functions	as in *Larsen et al (2016)* (uses feature differences in Discriminator as the auto-encoder reconstruction loss)				
Optimizer	Adam, learning rate = 0.0001				

Supplementary Table 1. Architecture of the VAE-GAN network. fc: fully connected; elu: exponential linear units
