# Supplementary material for: Reconstructing faces from fMRI patterns using deep generative neural networks
Source: Commun Biol. 2019 May 21;2:193. doi: 10.1038/s42003-019-0438-y (PMC6529435; doi:10.1038/s42003-019-0438-y)
Supplement: Supplementary file 3 — Description of Supplementary Data [file 42003_2019_438_MOESM3_ESM.docx]

**Description of Additional Supplementary Files**

**File Name**: Supplementary Data 1

**Description**: Source data used to produce Results Figures 4B-C, 5, 6 and 7 (Microsoft Excel format).
