# Supplementary material for: Reconstructing faces from fMRI patterns using deep generative neural networks
Source: Commun Biol. 2019 May 21;2:193. doi: 10.1038/s42003-019-0438-y (PMC6529435; doi:10.1038/s42003-019-0438-y)
Supplement: Supplementary file 4 — Reporting Summary [file 42003_2019_438_MOESM4_ESM.pdf]

nature research

Corresponding author(s): Rufin VanRullen (April 1st, 2019)

Reporting Summary

Nature Research wishes to improve the reproducibility of the work that we publish. This form provides structure for consistency and transparency in reporting. For further information on Nature Research policies, see Authors & Referees and the Editorial Policy Checklist.

Statistical parameters

When statistical analyses are reported, confirm that the following items are present in the relevant location (e.g. figure legend, table legend, main text, or Methods section).

n/a	Confirmed	
☐	☒	The exact sample size (n) for each experimental group/condition, given as a discrete number and unit of measurement
☒	☐	An indication of whether measurements were taken from distinct samples or whether the same sample was measured repeatedly
☐	☒	The statistical test(s) used AND whether they are one- or two-sided<br>*Only common tests should be described solely by name; describe more complex techniques in the Methods section.*
☐	☒	A description of all covariates tested
☐	☒	A description of any assumptions or corrections, such as tests of normality and adjustment for multiple comparisons
☐	☒	A full description of the statistics including central tendency (e.g. means) or other basic estimates (e.g. regression coefficient) AND variation (e.g. standard deviation) or associated estimates of uncertainty (e.g. confidence intervals)
☐	☒	For null hypothesis testing, the test statistic (e.g. F, t, r) with confidence intervals, effect sizes, degrees of freedom and P value noted<br>*Give P values as exact values whenever suitable.*
☒	☐	For Bayesian analysis, information on the choice of priors and Markov chain Monte Carlo settings
☒	☐	For hierarchical and complex designs, identification of the appropriate level for tests and full reporting of outcomes
☒	☐	Estimates of effect sizes (e.g. Cohen's d, Pearson's r), indicating how they were calculated
☐	☒	Clearly defined error bars<br>*State explicitly what error bars represent (e.g. SD, SE, CI)*

Our web collection on statistics for biologists may be useful.

Software and code

Policy information about availability of computer code

Data collection	Data collected with custom code written in Matlab R2013 with the Psychtoolbox extension. Stimuli generated with open-source Python3 code and Tensorflow 1.8 Libraries.
Data analysis	Analysis done with FreeSurfer, SPM12 for Matlab R2013, and with custom code written in Matlab R2013.

For manuscripts utilizing custom algorithms or software that are central to the research but not yet described in published literature, software must be made available to editors/reviewers upon request. We strongly encourage code deposition in a community repository (e.g. GitHub). See the Nature Research guidelines for submitting code & software for further information.

Data

Policy information about availability of data

All manuscripts must include a data availability statement. This statement should provide the following information, where applicable:

- Accession codes, unique identifiers, or web links for publicly available datasets
- A list of figures that have associated raw data
- A description of any restrictions on data availability

The full fMRI datasets for all four subjects (source dta: raw nifti files, event files and stimulus set) are available on OpenNeuro, an open data sharing and analysis

platform (https://openneuro.org/datasets/ds001761). The repository also contains the brain decoding models (SPM processed data, as well as Matlab code for producing latent vector estimates from fMRI data) as derivatives. The pre-trained VAE-GAN network with accompanying Python and TensorFlow source code is fully available on GitHub at: https://github.com/rufinv/VAE-GAN-celebA

Field-specific reporting

Please select the best fit for your research. If you are not sure, read the appropriate sections before making your selection.

☒ Life sciences ☐ Behavioural & social sciences ☐ Ecological, evolutionary & environmental sciences

For a reference copy of the document with all sections, see nature.com/authors/policies/ReportingSummary-flat.pdf

Life sciences study design

All studies must disclose on these points even when the disclosure is negative.

Sample size	Sample size (n=4) was determined according to standard practices in the field of fMRI decoding, where the emphasis is on obtaining reliable data for each subject (more than 12 hours of scanning per subject), and the effects are demonstrated indivdually for each subject, rather than over the group or population.
Data exclusions	No data were excluded.
Replication	Each of the experimental subjects can be considered as a replication of the analysis. With n=4, the main finding was independently replicated 3 times.
Randomization	The statistical design (for comparison between our method and prior state-of-the-art) is a within-subject design, therefore no subject randomization was necessary.
Blinding	Blinding was not relevant, since no randomization was applied.

Reporting for specific materials, systems and methods

Materials & experimental systems

n/a	Involved in the study	
☒	☐	Unique biological materials
☒	☐	Antibodies
☒	☐	Eukaryotic cell lines
☒	☐	Palaeontology
☒	☐	Animals and other organisms
☐	☒	Human research participants

Methods

n/a	Involved in the study	
☒	☐	ChIP-seq
☒	☐	Flow cytometry
☐	☒	MRI-based neuroimaging

Human research participants

Policy information about studies involving human research participants

Population characteristics	The four subjects were male lab members, aged 24-44.
Recruitment	Subjects were recruited for their ability and willingness to spend a combined 12 hours of recording in the scanner. This means that they were fMRI experienced subjects. It is not possible that the choice of subjects influenced the experiment outcome, since the exact same data was used for both decoding methods compared.

Magnetic resonance imaging

Experimental design

Design type	Event-related design
Design specifications	Each subject was tested in 8 scan sessions. Subjects performed between 10 and 14 face runs in each scan session. Each face run started and ended with a 6 s blank interval. Subjects were presented with 88 face stimuli. Each face was presented for 1s, followed by an inter-stimulus interval of 2s (i.e., the inter-trial interval was 3s). The faces subtended 8

degrees of visual angle, and were presented at the center of the screen. Ten test faces (five male and five female) were randomly interspersed among the 88 face stimuli on each run. On alternate runs a different group of 10 test faces was presented (i.e., 20 test faces per subject). Thirty null "fixation" trials were interspersed in each run during which, instead of the face stimulus, a fixation cross was presented on the screen. To keep subjects alert and encourage them to pay attention to the face stimuli, they were instructed to perform a "1-back" comparison task: press a button as fast as possible whenever the face image was identical to the immediately preceding face. In addition to the 88 face trials, there were 8 one-back trials in each run, and the repeated images were discarded from the brain decoder training procedure (described below). Additionally, whenever the sequence of face images was replaced by a large static gray square (lasting 12s) in the middle of the screen, subjects mentally imagined one specific face image that they had previously chosen among a set of 20 possible faces. For a given subject, only one face image was chosen and studied at length (outside the scanner, between scanning sessions 4 and 5), and then imagined repeatedly throughout scanning sessions 5-8. In odd (respectively even) scanning runs, a unique 12s imagery trial was introduced at the beginning (respectively, the end) of the run. Over the four experimental subjects, the number of recorded imagery trials ranged from 51 to 55 (mean 52). A 6 s blank period followed every imagery trial.

Behavioral performance measures: The main experimental measure is brain decoding accuracy, which is independent of subjects' behavioral performance.

Acquisition

Imaging type(s): functional

Field strength: 3T

Sequence & imaging parameters: Functional MRI data were collected on a 3T Philips ACHIEVA scanner (gradient echo pulse sequence, TR = 2 s, TE = 10 ms, 41 slices with a 32 channel head coil, slice thickness = 3 mm with 0.2 mm gap, in-plane voxel dimensions 3 x 3 mm). High-resolution anatomical images were also acquired per subject (1×1×1mm voxels, TR = 8.13 ms, TE = 3.74 ms, 170 sagittal slices).

Area of acquisition: The slices were positioned to cover the entire temporal and occipital lobes.

Diffusion MRI: ☐ Used ☒ Not used

Preprocessing

Preprocessing software: fMRI data were processed with SPM 12 (https://www.fil.ion.ucl.ac.uk/spm/software/spm12/). For each participant data from each scan session were slice-time corrected and realigned separately. Then each session was co-registered to the T1 scan from the second MRI session.

Normalization: The data were not normalized or smoothed.

Normalization template: The data were not normalized or smoothed.

Noise and artifact removal: Motion parameters were entered as nuisance regressors.

Volume censoring: No volume censoring was applied.

Statistical modeling & inference

Model type and settings: The onset and durations of each trial (fixation, training-face, test-face, one-back, or imagery) were entered into a general linear model (GLM) as regressors and convolved with a hemodynamic response function. Optionally, the 1024 latent vectors (either from the VAE-GAN or the PCA model) of the training face images could be modeled as parametric regressors.

Effect(s) tested: The 1024 parametric regressors (face parameters) for each voxel were entered in a multi-voxel pattern decoding algorithm.

Specify type of analysis: ☒ Whole brain ☐ ROI-based ☐ Both

Statistic type for inference (See Eklund et al. 2016): The relevant measure was pattern decoding accuracy. No voxelwise statistics, or voxelwise correction for multiple comparisons was required.

Correction: The relevant measure was pattern decoding accuracy. No voxelwise statistics, or voxelwise correction for multiple comparisons was required.

Models & analysis

n/a	Involved in the study
☒	☐ Functional and/or effective connectivity
☒	☐ Graph analysis
☐	☒ Multivariate modeling or predictive analysis

Multivariate modeling and predictive analysis: We trained a simple brain decoder (linear regression) to associate the 1024-D latent representation of face images (obtained by running the image through the "Encoder", as described in Figure 1, or using a PCA

transform as described above and in Supplementary Figure S1) with the corresponding brain response pattern, recorded when a human subject viewed the same faces in the scanner. This procedure is illustrated in Figure 2A. Each subject saw more than 8,000 faces on average (one presentation each) in a rapid event-related design, and we used the VAE-GAN latent dimensions (or the image projection onto the first 1024 PCs) as 1024 parametric regressors for the BOLD signal (see fMRI analysis section above). The linear regression performed by the SPM GLM analysis thus produced a weight matrix W (1025 by nvoxels dimensions, with a constant "bias" term added to the 1024 latent dimensions, and where nvoxels is the number of voxels in the brain region-of-interest) optimized to predict brain patterns in response to the training face stimuli.
To use this brain decoder in the "testing phase", we simply inverted the linear system, as illustrated in Figure 2B. We presented 20 novel test faces to the same subjects, which had not been seen in the training phase. Each test face was presented on average 52.8 times (range across subjects: [45.4-55.8], randomly interleaved with the training face images) to increase signal-to-noise ratio. The resulting brain activity patterns were simply multiplied by the transposed weight matrix WT (nvoxels by 1025 dimensions) and its inverse covariance matrix to produce an estimate of the 1024 latent face dimensions (in addition to an estimate of the bias term, which was not used further). We then used the Generator network (as illustrated in Figure 1A) to translate the predicted latent vector into a reconstructed face image. For the baseline PCA model, the same logic was applied, but the face reconstruction was obtained via inverse PCA of the decoded 1024-D vector.
